# PP2A inhibition with LB100 enhances cisplatin cytotoxicity and overcomes cisplatin resistance in medulloblastoma cells

**DOI:** 10.18632/oncotarget.6970

**Published:** 2016-01-21

**Authors:** Winson S. Ho, Michael J. Feldman, Dragan Maric, Lauren Amable, Matthew D. Hall, Gerald M. Feldman, Abhik Ray-Chaudhury, Martin J. Lizak, Juan-Carlos Vera, R. Aaron Robison, Zhengping Zhuang, John D. Heiss

**Affiliations:** ^1^ Surgical Neurology Branch, National Institute of Neurological Disorders and Stroke, National Institutes of Health, Bethesda, MD 20892; ^2^ NINDS Flow Cytometry Core Facility, National Institute of Neurological Disorders and Stroke, National Institutes of Health, Bethesda, MD 20892; ^3^ National Institute on Minority Health and Health Disparities, National Institutes of Health, Bethesda, MD 20892; ^4^ National Center for Advancing Translational Sciences, National Institutes of Health, Rockville, MD 20850, USA; ^5^ Center for Drug Evaluation and Research, Food and Drug Administration, Silver Spring, MD 20993; ^6^*In Vivo* NMR Center, National Institute of Neurological Disorder and Stroke, National Institutes of Health, Bethesda, MD 20892; ^7^ School of Medicine, Tulane University, New Orleans, LA 70112; ^8^ Division of Neurosurgery, Children's Hospital Los Angeles, University of Southern California, Los Angeles, CA 90027

**Keywords:** medulloblastoma, PP2A, LB100, STAT3, cisplatin

## Abstract

The protein phosphatase 2A (PP2A) inhibitor, LB100, has been shown in pre-clinical studies to be an effective chemo- and radio-sensitizer for treatment of various cancers. We investigated effects associated with LB100 treatment alone and in combination with cisplatin for medulloblastoma (MB) *in vitro* and *in vivo* in an intracranial xenograft model. We demonstrated that LB100 had a potent effect on MB cells. By itself, LB100 inhibited proliferation and induced significant apoptosis in a range of pediatric MB cell lines. It also attenuated MB cell migration, a pre-requirement for invasion. When used in combination, LB100 enhanced cisplatin-mediated cytotoxic effects. Cell viability in the presence of 1 uM cisplatin alone was 61% (DAOY), 100% (D341), and 58% (D283), but decreased with the addition of 2 μM of LB100 to 26% (DAOY), 67% (D341), and 27% (D283), (*p* < 0.005). LB100 suppressed phosphorylation of the STAT3 protein and several STAT3 downstream targets. Also, LB100 directly increased cisplatin uptake and overcame cisplatin-resistance *in vitro*. Finally, LB100 exhibited potent *in vivo* anti-neoplastic activity in combination with cisplatin in an intracranial xenograft model.

## INTRODUCTION

Medulloblastoma (MB) is the most common malignant brain tumor in the pediatric population, accounting for about 18% of all central nervous system (CNS) tumors in childhood [1]. Current standard therapy involves maximal surgical resection followed by craniospinal radiotherapy and adjuvant chemotherapy [2], which consists of cisplatin, vincristine, and either lomustine or cyclophosphamide. This treatment paradigm can achieve a survival rate as high as 75–85% [3], but patients often suffer from treatment related long-term sequelae ranging from neurologic deficit and endocrinopathy to neurocognitive impairment [4, 5]. For 20 to 30% of patients with disease recurrence after radiation and chemotherapy, prognosis is particularly poor with median survival of 26.8 months [6]. Treatment options are limited and ineffective as recurrent tumors are often resistant to chemotherapy and radiation. Therefore, there is considerable interest in developing novel therapeutic approaches to MBs that are less toxic and more effective in overcoming resistance in chemotherapy. Recent molecular classification of MBs into four genetic sub-groups (Wnt, SHH, Group3 and Group 4) [7] has spurred interest in molecular-pathway targeted therapies [8]. However, chemotherapy and radiation remain the mainstay therapeutic option for MBs. Sensitizing tumors to chemo-radiation by inhibiting PP2A activity with the small molecule compound, LB100, is a novel approach that was effective in a number of preclinical studies for several different cancers [9].

Protein phosphatase 2A (PP2A) is a ubiquitous serine/threonine phosphatase that is implicated in a broad array of regulatory cellular functions including cell survival, apoptosis, mitosis and DNA-damage response [10]. PP2A is considered a tumor suppressor gene and PP2A inhibition has been associated with tumorigenesis [11]. However, there is mounting evidence that inhibition of PP2A in a range of tumor types potently sensitizes them to radiation and chemotherapy [9]. LB100 is a small molecule PP2A inhibitor derived from the natural compound cantharadin but with a significantly more favorable toxicity profile [12]. It is currently in phase I clinical trial for treatment of adult solid tumors [13]. Previous preclinical studies have shown that LB100 is an effective chemo-sensitizer against glioblastoma [12], ovarian cancer [14], sarcoma [12, 15] and hepatocellular carcinoma [16]. It is also an effective radio-sensitizer against glioblastoma [17], nasopharyngeal carcinoma [18] and pancreatic cancer [19]. Given the ubiquity of PP2A, the inhibition of LB100 likely has multiple downstream effects. In aggregate, evidence so far suggests that PP2A inhibition with LB100 can result in down regulation of DNA-damage response [12, 14, 19], abrogation of cell cycle checkpoint [12, 17], increase in HIF dependent tumor angiogenesis [16] and induction of cellular differentiation by inhibition of N-CoR complex formation [12].

The activity of Signal Transducer and Activator of Transcription 3 (STAT3) is widely known to play a crucial role in the tumorigenesis of multiple different cancers [20]. Previous studies have shown that MB has a high level of expression and constitutive activation of STAT3 [21–23]. Inhibition of the STAT3 axis has been shown to be an effective therapeutic strategy *in vitro* and *in vivo* against MB by conferring direct inhibitory effect [23–26] or by enhancing chemo- or radio-sensitivity [27, 28]. Inhibition of PP2A has previously been shown to inactivate STAT3 activity by inducing serine-727 phosphorylation [29, 30] and conversely down-regulating Tyr-705 phosphorylation [31], which is the key mediator of STAT3 transcriptional activity. We thereby hypothesize that LB100 could exert an antineoplastic effect on MB cells via down regulation of STAT3, a novel mechanism not previously reported for LB100.

This study was designed to provide preclinial data for the potential use of LB100 in conjunction with cisplatin in the treatment of MBs. LB100 and cisplatin are administered to a range of pediatric MB cell lines and an MB intracranial xenograft. The effects of LB100 on phosphorylation of the STAT3 protein and several STAT3 downstream targets are measured to provide mechanistic information about LB100 action in MB cells. The effect of LB100 on cisplatin uptake and resistance is also investigated.

## RESULTS

### MB cell line sensitivity to LB100 and cisplatin

**Figure 1:**
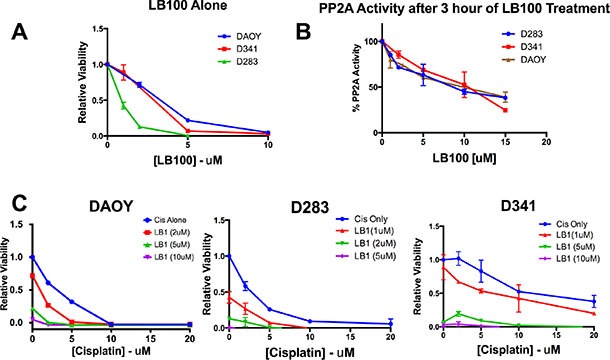
LB100 reduces MB cells viability, PP2A activity and enhances cisplatin mediated cytotoxicity *in vitro* Three MB cell lines cell lines DAOY, D283 and D341 were used *in vitro*. XTT assays were performed after 48 hours of treatment. (**A**) Increasing concentrations of LB100 reduces cell viability in a dose dependent manner. (**B**) PP2A activity was measured based on a phosphatase assay and expressed as a percentage of control. After 3 hours of LB100 treatment, PP2A activity was decreased in a dose-dependent fashion in all three cell lines. (**C**) Cells were treated with various combinations of LB100 and cisplatin. Increasing LB100 enhances cisplatin-mediated cytotoxicity. LB100 was given as pre-treatment 3 hours prior to giving cisplatin. Data are represented as mean +/− SEM.

To assess the sensitivity of MB cells to LB100 and cisplatin *in vitro*, three human MB cell lines were used: DAOY, D341 and D283, which belong to the SHH, group 3 and group 4 subtypes of MB respectively [32, 33]. LB100 demonstrated cytotoxicity against all three-cell lines. A dose-response curve using XTT assays after 48 hours of drug treatment demonstrated IC50 of 2.9, 1.9 and 0.9 μM for DAOY, D341 and D283 cells respectively (Figure 1A).

To assess the effect of LB100 on PP2A function in MB cells, we measured PP2A activity 3 hours after LB100 treatment. LB100 caused a concentration dependent decrease in PP2A activity in all three MB cell lines (Figure 1B). LB100 significantly enhanced cisplatin-mediated cytotoxicity (Figure 1C) in a dose dependent manner. Relative viability significantly decreased from 61% to 26% with 2 μM of LB100 in DAOY and from 100% to 67% and 58% to 27% with 1 μM of LB100 in D341 and D283 cells respectively (*p* < 0.005).

### LB100 induces anti-proliferative and pro-apoptotic effects in MB cell lines

**Figure 2:**
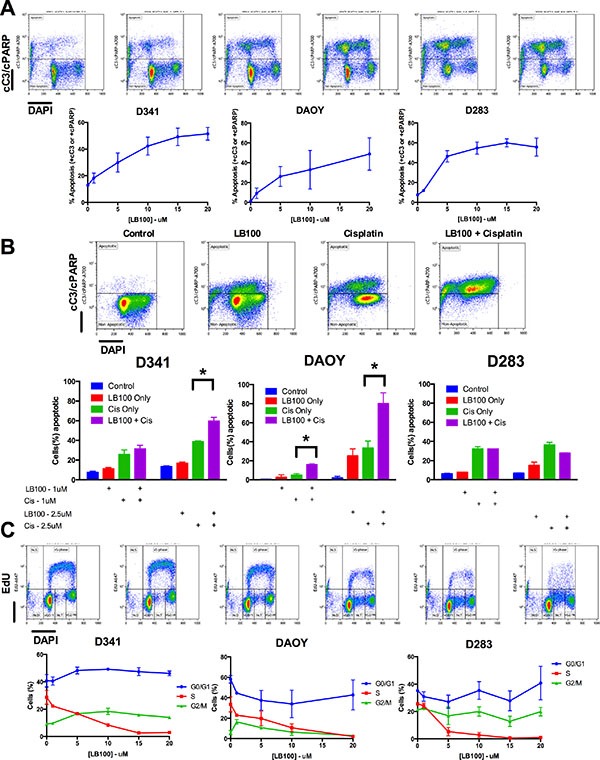
Analysis of LB100 induced apoptosis and cell cycle changes DAOY and D341 cells were used after 48 hours of drug treatment. (**A**) Double staining with cC3/cPARP-DAPI and flow cytometry analysis was performed to determine rate of apoptosis with increasing concentration of LB100. Quantification of the flow cytometry data shows a dose-dependent increase in apoptosis with LB100 treatment. (**B**) Rate of apoptosis was compared between LB100, cisplatin alone and in combination. Two concentrations – 1 μM and 2.5 μM – of each drug and their combinations were tested. Statistically significant differences are marked by an asterisk (*p < 0.05) (**C**) Cell cycle analysis was performed with increasing concentration of LB100 treatment. Double staining with EdU-DAPI and flow cytometry analysis identifies G0/1, S and G2/M phases. Cell cycle distribution of the three phases with different concentration of LB100 treatment is represented for each cell line. Data are represented as mean +/− SEM.

The effect of LB100 on apoptosis was examined using flow cytometry after 48 hours of drug treatment in DAOY and D341 cell lines. Apoptotic cells were labeled using antibodies targeting cleaved caspase-3 (cC3) and cleaved poly ADP ribose polymerase (cPARP), both widely used as apoptotic markers. Apoptosis was induced in a dose-dependent manner (Figure 2A). In DAOY cells, apoptosis increased from 1% in control to 49% with 20 μM LB100. In D341, apoptosis increased from 13% in control to 51% with 20 μM LB100.

The effect of combining cisplatin with LB100 on the induction of apoptosis was also examined (Figure 2B). Using two different concentrations (1 μM or 2.5 μM) of cisplatin and LB100 alone or in combination, apoptosis was assessed after 48 hours of drug treatment. In DAOY cells, LB100 and cisplatin combination significantly increased apoptosis compared to either drug alone in both concentrations. At the lower concentration of 1 μM, the LB100 and cisplatin combination induced apoptosis in 16% compared to 3.8% (*P* < 0.05) and 0.8% (*P* < 0.05) of cells with cisplatin and LB100 alone respectively. At the higher concentration of 2.5 μM, the combination induced apoptosis in 80% compared to 33.3% (*P* < 0.05) and 25.1% (*P* < 0.05) of cells with cisplatin and LB100 alone respectively. In D341 cells, the LB100-cisplatin combination significantly increased apoptosis at concentration of 2.5 μM, with apoptosis occurring in 60% of cells with the combination treatment compared to 38.6% (*P* < 0.01) in cisplatin and 16.8% (*P* < 0.01) in LB100 alone. However, in D283 cells, the combination of LB100 and cisplatin did not significantly enhance apoptosis.

To elucidate the effect of LB100 on cell cycle of MB cells, cell cycle analysis with flow cytometry was performed after 48 hours of treatment with increasing concentration of LB100 in DAOY and D341. Cells were first gated to exclude the apoptotic cC3^+^/cPARP^+^ population and the remaining non-apoptotic cells were then assayed for cell cycle analysis (Figure 2C). In both cell lines, a low concentration of LB100 induced G2/M arrest. In DAOY, the proportion of cells in G2/M increased significantly from 5.8% to 17.3% (*P* < 0.05) with 1 μM of LB100. In D341, cells in G2/M also increased significantly from 9.1% to 16.6% at 5 μM of LB100. This effect plateaued or diminished at higher concentration. In addition, there was a dose-dependent decrease in S-phase at higher concentration of LB100 treatment. The % cells in S phase decreased from 33.6 to 2.1% and 28.7 to 2.9% from control to 20 μM of LB100 in DAOY and D341 cells respectively.

### LB100 slowed MB cell migration

**Figure 3:**
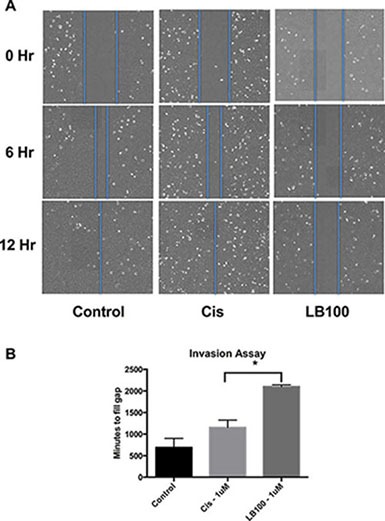
LB100 inhibits MB cell motility and migration Culture insert acting as a barrier between two wells plated with DAOY cells were removed leaving a 500 um gap for cells to migrate. Cells were treated with vehicle control (PBS), LB100 or cisplatin at 1 μM. Serial time-lapse images were taken every 15 mins for 48 hours. Representative images for each condition taken at 6 and 12 hours were shown. The time it takes for the cells to populate the 500 um gap was then compared. Statistically significant differences are marked by an asterisk (*p ≤ 0.05). Data are represented as mean +/− SEM.

Tumor cell mobility is thought to play a critical role in invasion and metastasis [24, 25], DAOY cells are known for their migratory properties, and were used to investigate whether LB100 can inhibit cell mobility in a wound-healing assay (Figure 3A–3B). After removal of a culture-insert leaving a 500 μm gap between the plated cells, the cells were treated with control, 1 μM LB100 or 1 μM cisplatin (< = IC25). Serial images of the tumor cells were collected over the next 48 hours. Representative images at 6- and 12-hours following treatment are shown in Figure 3A. The amount of time it took for the cells to invade into the 500 μm gap was significantly longer with LB100 treatment compared to cisplatin or control (11.5 hours control, 19.3 hours in cisplatin and 35 hours in LB100, *P* < 0.05). This result strongly suggests that LB100 profoundly affects the migratory property of tumor cells potentially impairing their ability to invade and metastasize.

### LB100 treatment was associated with downregulated STAT3 phosphorylation at tyrosine 705 and increased phosphorylation at serine 727

**Figure 4:**
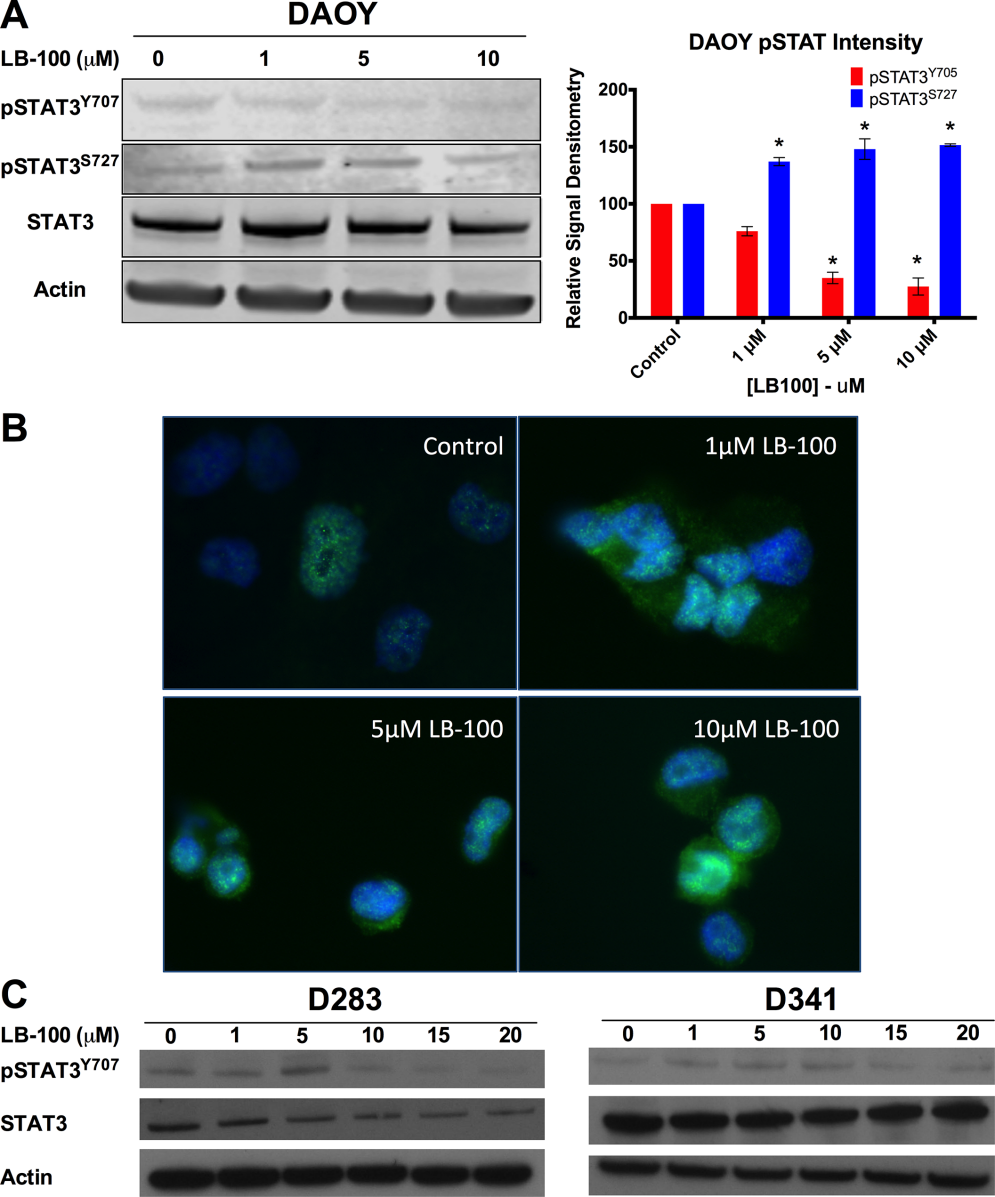
LB100 inhibits STAT3 activation (**A**) Western blot with densitometry analysis demonstrated decrease in tyrosine-705 phosphorylation and increase in serine-727 phosphorylation with increasing concentration of LB100 treatment. Statistically significant differences compared to control are indicated by an asterisk (*p < 0.01). (**B**) Immunocytochemical staining for STAT3 was performed after 4 hours of LB100 treatment at the indicated concentrations. There was an increase in cytoplasmic localization of STAT3 consistent with inactivation of STAT3. (**C**) Western blots for STAT3Tyr-705 in D283 and D341 with increasing LB100 concentration showing a similar pattern of decrease in STAT3Tyr-705 activation as DAOY cells. Data are represented as mean +/− SEM.

Immunoblotting and densitometry analyses in DAOY cells were used to examine if PP2A inhibition by LB100 could down-regulate STAT3 activation by inducing serine-727 phosphorylation and conversely decreasing tyrosine-705 phosphorylation. Phosphorylation increased at serine 727 and decreased at tyrosine 705 in a dose-dependent manner after 24-hour of LB100 treatment in DAOY cells (Figure 4A). Serine 727 phosphorylation was increased by 31% to 51% and tyrosine 705 phosphorylation was inhibited by 24% to 73% compared to control with 1 μM to 10 μM of LB100 treatment. Two-sample equal variance *t* test of the normalized data showed that the increase in serine 727 phosphorylation compared to control was significant for all three concentrations (*p* < 0.01) while the decrease in tyrosine phosphorylation was significant for 5 and 10 um compared to control. A similar change in pattern of phosphorylation status was seen as early as four hours after treatment (data not shown). Total STAT3 level was unchanged in treated DAOY cells. To further confirm inactivation of STAT3, the subcellular distribution of STAT3 was examined using immunocytochemistry staining. As shown in Figure 4B, there was a concentration dependent change in subcellular distribution of STAT3 from nuclear to cytoplasmic localization with LB100 treatment, corresponding to inactivation of STAT3. To confirm if inactivation of STAT3 is a common mechanism of LB100 treatment, immunoblotting was performed on D283 and D341 cells (Figure 4C). Decreased tyrosine 705 phosphorylation was similarly observed but only at higher LB100 concentrations.

### LB100 inhibited protein expression of downstream target of STAT3

**Figure 5:**
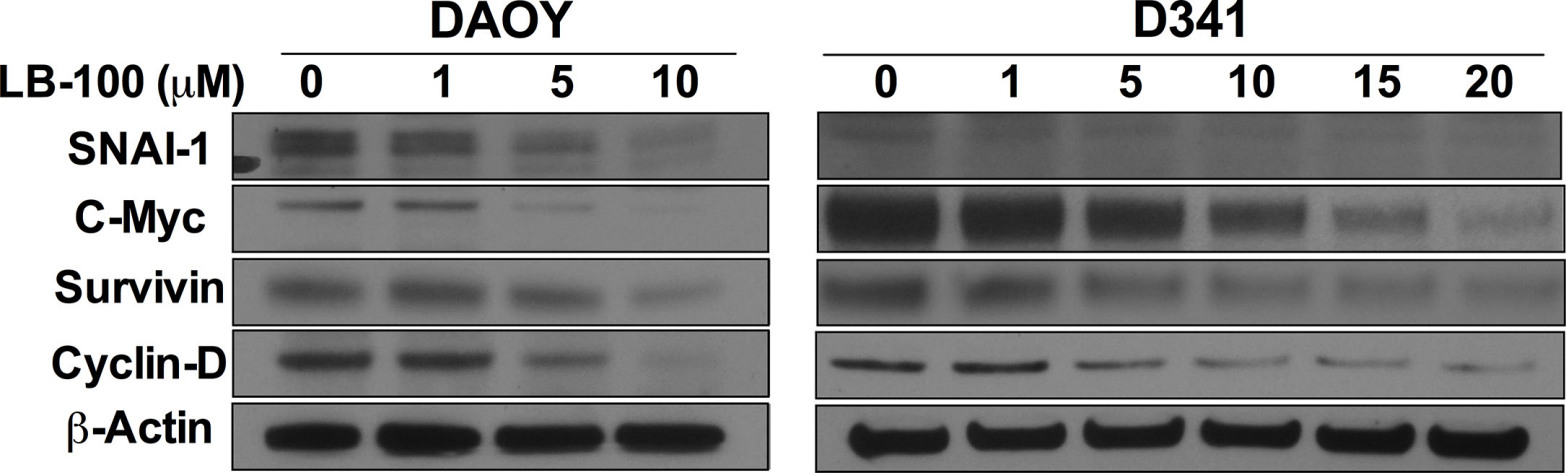
LB100 down-regulates expression of STAT3 target genes Western blot analysis of DAOY and D341 cells treated with increasing LB100 concentration demonstrates dose-dependent decrease in STAT3 target genes, including SNAIL, c-myc, survivin and cyclin D.

To further confirm down-regulation of STAT3, the protein expression of four STAT3 downstream targets, including cyclin D, c-myc, survivin and SNAIL, were examined with immunoblotting in DAOY and D341 cells (Figure 5). All four targets (cyclin D, c-myc, survivin and SNAIL) were significantly inhibited in a dose-dependent manner with LB100 treatment.

### LB100 increased cisplatin uptake and down-regulated MRP1

**Figure 6:**
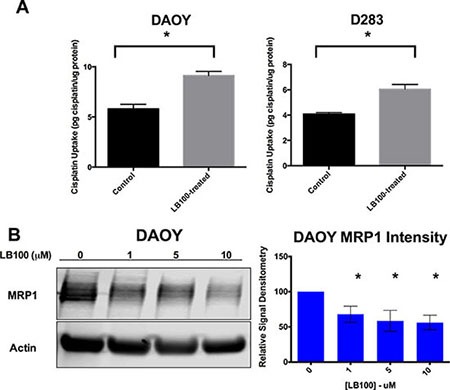
LB100 decreases cisplatin uptake and is associated with down-regulation of MRP1 (**A**) DAOY and D283 cells were treated with 2.5 μM of LB100 for 24 hours followed by 5 μM cisplatin for 1 hour. ICP-MS was then used to measure the total whole cell cisplatin level. LB100 treated cells showed a significant increase in cisplatin level suggesting PP2A inhibition affect cisplatin influx or efflux. (**B**) Western blot with densitometry was performed on DAOY cells demonstrating LB100 treatment resulted in a significant decrease in expression of MRP1, a known ATP-binding cassette (ABC) drug transporter involved in cisplatin efflux. Statistically significant differences compared to control are marked by an asterisk (*p ≤ 0.05). Data are represented as mean +/− SEM.

Whole-cell platinum levels were measured by Inductively Coupled Plasma-Mass Spectrometry (ICP-MS) after one hour of cisplatin treatment with or without LB100 (Figure 6A). In both DAOY and D283 cells, LB100 treatment significantly increased whole-cell platinum level, suggesting that increase in cisplatin sensitivity by PP2A inhibition was in part due to direct increase in cisplatin uptake. Cisplatin uptake increased by 57% and 48% in DAOY and D283 cells respectively. In DAOY cells, immunoblotting showed LB100 treatment for 24 hours decreased MRP1 expression by 32 to 44% from 1 μM to 10 μM of drug treatment (Figure 6B), suggesting that PP2A inhibition-mediated cisplatin sensitivity was associated with MRP1 down-regulation [35, 37]. It has been shown that in atypical teratoid/rhabdoid tumors, cisplatin-resistance could result from activation of STAT3/Snail axis and up-regulation of MRP1 [34], a ATP-binding cassette (ABC) drug transporter that is involved in development of multi-drug resistance in different cancers [35]. So we examined whether PP2A inhibition-mediated cisplatin sensitivity is associated with MRP1 down-regulation.

### LB100 overcame cisplatin-resistance *in vitro*

**Figure 7:**
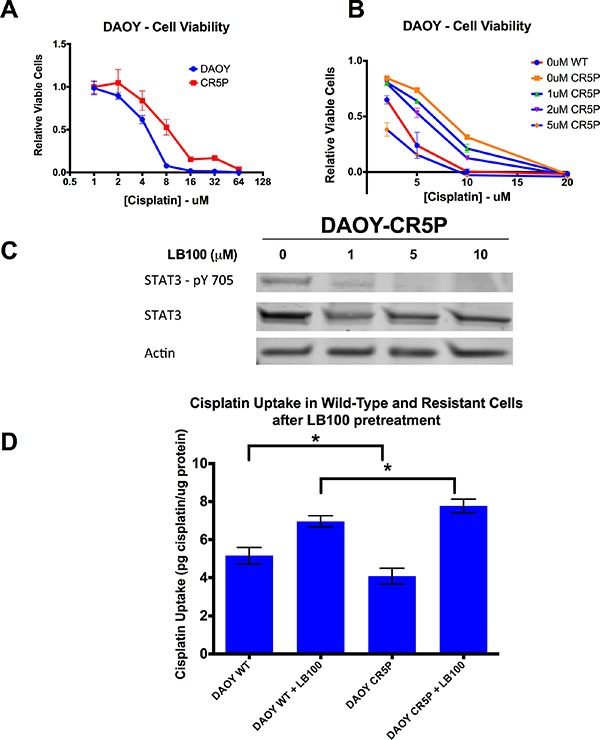
LB100 overcomes *in vitro* cisplatin resistance, decreases STAT3 activation and increases cisplatin uptake in cisplatin-resistant cells (**A**) DAOY cells were treated with increasing pulses of cisplatin for up to four months until a stably resistant cell line was generated (DAOY-CR5). Using XTT assay, the cisplatin IC50 after 48 hours of cisplatin treatment was determined. DAOY-CR5 has an IC50 of 7.4 μM compared to 4.5 μM in wild-type parental DAOY cells (DAOY-WT). (**B**) XTT assay comparing cisplatin sensitivity of DAOY-WT and DAOY-CR5 with varying concentration of LB100 demonstrated that at concentration between 2 and 5 μM, the cisplatin–resistance of DAOY-CR5 was completely abolished by LB100. (**C**) Western blot analysis showed that LB100 was able to down-regulate STAT3 activation independent of cisplatin resistance. (**D**) DAOY-WT and DAOY-CR5 cells were treated with 5 μM of LB100 for 4 hours and ICP-MS was performed after 1 hour of cisplatin treatment to measure total whole cell cisplatin level. DAOY-CR5 cells showed a significant decrease in cisplatin uptake at baseline. LB100 treatment of DAOY-CR5 cells was able to reverse this decrease and enhanced cisplatin uptake compared to LB100-treated DAOY WT cells, strongly suggesting that LB100 could overcome cisplatin resistance by increasing cisplatin uptake. Statistically significant differences are marked by an asterisk (*p ≤ 0.05). Data are represented as mean +/− SEM.

To examine whether LB100 can overcome cisplatin resistance, we established a cisplatin-resistant DAOY cell line (termed DAOY-CR5P). Treating DAOY cells with pulses of increasing concentrations of cisplatin over 4 months resulted in generation of a stably resistant cell line with a cisplatin IC50 of 7.4 μM compared to 4.5 μM in the parental wild type (DAOY-WT) (Figure 7A). In XTT cell viability assays performed to compare cisplatin sensitivity of DAOY-WT to DAOY-CR5P with increasing concentration of LB100 (Figure 7B), resistance to cisplatin was completely abolished with LB100 between 2 and 5 μM. Low LB100 concentrations profoundly decreased tyrosine-705 phosphorylation (STAT3 activation) in the cisplatin resistant cell line, similar to DAOY-WT (Figure 7C). ICP-MS comparing platinum levels in WT and CR5P cells (Figure 7D) showed that the resistant cells had reduced cisplatin uptake compared to DAOY-WT. LB100 treatment increased cisplatin uptake more in DAOY-CR5P than in DAOY-WT.

### LB100, combined with cisplatin, inhibited growth of the MB intracranial xenograft

**Figure 8:**
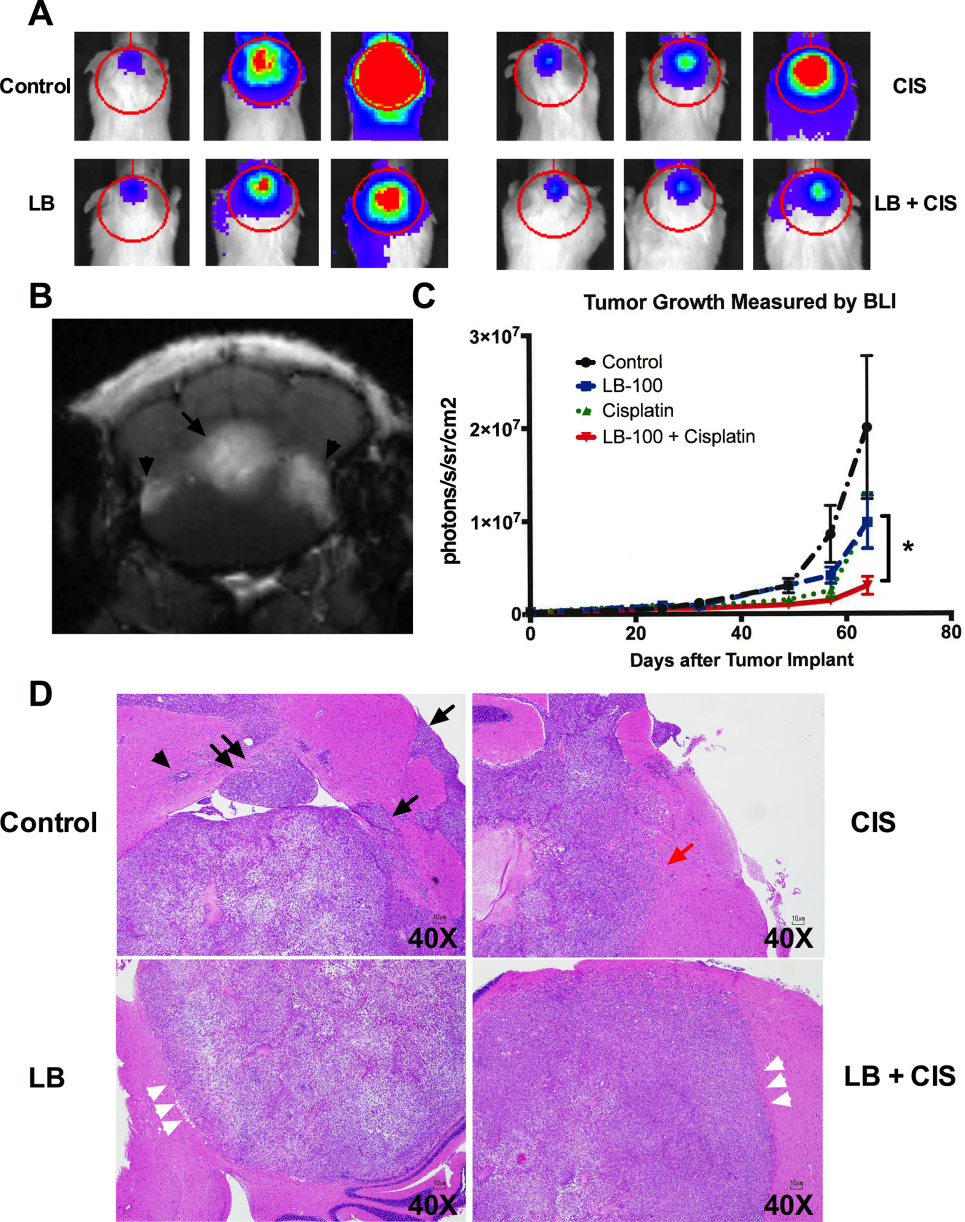
LB100 in combination with cisplatin significantly decreases tumor burden *in vivo* After implantation of FLuc-expressing DAOY cells, mice were randomized into four treatment groups [vehicle (PBS) control (*n* = 7), LB100 (1.5 mg/kg; *n* = 6), cisplatin (1.5 mg/kg; *n* = 5), and LB100 (given 1 hour before cisplatin) + cisplatin (*n* = 5)]. After completion of treatment, tumor growth was monitored with whole body bioluminescence (BLI) every 1-2 week. (**A**) Representative BLI images were shown for a mouse in each treatment group on POD 10, 39 and 64. (**B**) MRI of the brain with gadolinium contrast of a representative mouse in the control group prior to sacrifice was performed to characterize the xenograft tumor. The MRI demonstrated the xenograft closely recapitulate the findings in human MBs, with a homogenously enhancing lesion in the cerebellum, invasion of the fourth ventricle (arrow) and leptomeningeal spread (arrow heads). (**C**) Tumor burden was assessed with serial BLI. By POD64, the mean bioluminescent signal was significantly lower in the combination group than the cisplatin group. Statistically significant differences are marked by an asterisk (*p ≤ 0.05). Data are represented as mean +/− SEM. (**D**) Histologic features of tumors in the cerebellum at survival endpoint of the animals were examined. In control group, tumors exhibited nodular invasion of the parenchyma and pia (black arrows), perivascular spread (black arrowhead) and intraventricular involvement (double arrows). In the cisplatin treated group, tumors were less invasive in appearance but continued to show parenchymal invasion at the border of the tumors (red arrow). In both LB100 treated groups, the tumors were markedly more circumscribed with a clear demarcating border between tumor and parenchyma (white arrowheads).

To confirm the *in vitro* anti-neoplastic activity of LB100 *in vivo*, we tested it alone and in combination with cisplatin in an intracranial orthotopic mouse model. For this purpose, we transduced DAOY cells with a lentivirus to stably express luciferase. Cells (2.5 × 10^5^) were implanted into the right cerebellum of immunocompromised SCID mice, Cell implantation resulted in cerebellar tumor formation detectable with bioluminescent imaging (BLI). The MB xenograft recapitulated the tumor morphology in human MB, as shown by MRI of the brain (Figure 8B). Tumors were clearly delineated, contrast-enhancing lesions extending into the fourth ventricle and showing signs of leptomeningeal seeding.

Animals with established tumors at post-operative day (POD) 10 (detected by BLI) were then randomized into four groups: [vehicle (PBS) control (*n* = 7), LB100 (1.5 mg/kg; *n* = 6), cisplatin (1.5 mg/kg; *n* = 5), and LB100 (given 1 hour before cisplatin) + cisplatin (*n* = 5)]. At the time of randomization, the baseline mean bioluminescence signals were 2.4 × 10^5^, 2.4 × 10^5^, 3.1 × 10^5^ and 2.7 × 10^5^ photons/s/sr/cm^2^ for control, LB100, cisplatin and LB plus cisplatin groups respectively. Ordinary one-way ANOVA test showed that there was no difference in bioluminescence signal between the four groups at the time of treatment initiation (*p* = 0.64). Treatment was administered via i.p injections every other day (QOD) starting on POD11 for a total of 6 treatments. During treatment, mice were given saline hydration IP to prevent dehydration and to offset cisplatin-mediated nephrotoxicity. No significant toxicity was observed in any of the treatment groups. The combination of LB100 and cisplatin significantly decreased tumor burden by POD64 (Figure 8A and 8C), with bioluminescent signal of 3 × 10^6^ photons/s/sr/cm^2^ for the combination group compared to 1 × 10^7^ photons/s/sr/cm^2^ (*p* < = 0.05) for cisplatin alone. At the survival endpoint, the tumors in the cerebellum were examined histologically (Figure 8D). In the control group, tumors exhibited nodular invasion of the parenchyma and pia, perivascular spread and intraventricular involvement. In the cisplatin treated group, tumors were less invasive in appearance but continued to show parenchymal invasion at the border of the tumors. In both LB100 treated groups, the tumors were markedly more circumscribed with a clear border demarcating the tumor and parenchyma. This result confirms the *in vitro* finding of decreased invasiveness of LB100 treated tumors *in vivo*. This observation suggests that there is a lasting effect of the drug because the histology was examined many weeks after completion of drug treatment.

## DISCUSSION

MB is the most common malignant brain tumor in the pediatric population. Standard treatment consisting of maximal surgical resection, chemotherapy and craniospinal radiation confers long-term survival in 60–85% of patients depending on various risk factors [3]. However, therapy-related co-morbidity, especially long-term cognitive impairment, is debilitating in young children. The options for recurrent and metastatic disease are often limited and inefficacious. Therefore, novel therapeutic strategies to augment standard cytotoxic therapy are urgently needed. LB100 is a competitive inhibitor of PP2A that sensitizes to chemotherapy and radiation in other cancers [12, 14, 19]. Pre-clinical studies have indicated that LB100-mediated PP2A inhibition is an effective strategy for radiation- and chemo-sensitization in various cancers [9]. In this study, we showed that LB100 has a potent effect on MB cells. By itself, LB100 inhibited proliferation and induced significant apoptosis in a range of pediatric MB cell lines. It also attenuated MB cell migration, a requirement for invasion. When used in combination, LB100 enhanced cisplatin-mediated cytotoxicity *in vivo*. This is the first study showing effectiveness of LB100 in an intracranial xenograft model. Prior studies of LB100 in glioblastoma [12, 17] showed effectiveness against subdermal but not intracranial xenografts. In contrast, we demonstrated that LB100 has significant *in vivo* chemo-sensitizing activity against intracranial MB, a densely contrast-enhancing lesion with an impaired blood brain barrier.

Prior studies of LB100 have focused on PP2A-mediated down regulation of the DNA-damage response [12, 14, 19] and abrogation of cell cycle checkpoints [12, 17] resulting in mitotic catastrophe and cell death. However, previous studies showing STAT3 overexpression in MB [21–23] and PP2A inhibition-mediated inactivation of STAT3 in T-cells [29] led us to hypothesize down-regulation of STAT3 as another mechanism of LB100 action. Our experimental findings confirmed that LB100 inhibited STAT3-activation in a dose dependent manner across multiple MB cell lines. This inhibition was associated with increased serine-727 phosphorylation, which is modulated by PP2A activity [29] and has been shown to conversely regulate the primary activation phosphorylation site in tyrosine-705 [31]. Consistent with previous reports on the effect of STAT3 inhibition, we have shown that LB100 treatment of MB cells increases apoptosis, decreases cellular proliferation and decreases cellular mobility on heal assay [24, 25]. Our results suggest that at least in STAT3-overexpressing tumors, such as MB, inhibition of STAT3 may be an additional downstream mechanism of PP2A inhibition contributing to its anti-neoplastic effect. To further suggest that STAT3 inactivation is biologically significant in MBs, several STAT3 downstream targets with known prognostic and mechanistic importance were also down regulated, including survivin, c-myc and SNAIL. Survivin is an anti-apoptotic protein that has been shown to be an independent negative prognostic marker in MB [36]. C-myc amplification is associated with group 3 MB [37] conferring the worst clinical prognosis [7]. SNAIL is a known mediator of epithelial-to-mesenchymal transition (EMT) under STAT3 transcriptional control [34], associated with enhanced cell invasiveness, stem-cell like characteristics, and apoptotic resistance [38].

Interestingly, we have also shown an increase in total cisplatin uptake with LB100 treatment on ICP-MS. This suggests that PP2A inhibition-mediated enhancement of cisplatin sensitivity could be at least partially related to a direct increase in cisplatin uptake. The most likely explanation for increased cisplatin uptake is alteration of cisplatin influx and/or efflux through known cisplatin transporters. Indeed we have shown that MRP1, a known multidrug resistant ATPase, is down regulated with LB100 treatment. A prior study demonstrated that MRP1 is activated by STAT3 and is associated with cisplatin resistance in atypical teratoid/rhabdoid tumor cells [34]. However, it is uncertain if STAT3 or MRP1 down-regulation could explain the increase in cisplatin uptake observed in our study because the effect was observed as early as fours after treatment, too soon for down stream changes of STAT3 inhibition. It is more likely that a phosphorylation event, possibly mediated by PP2A, took place to alter cisplatin permeability. To our knowledge, none of the known cisplatin transporters are regulated by phosphorylation. We measured intracellular copper level with ICP-MS in control and LB100 treated cells and found no difference in copper uptake (data not shown). We thereby ruled out CTR1, a copper transport protein known to play a significant role in cellular cisplatin uptake, as being involved in this LB100-mediated increase in cisplatin uptake. Future studies to elucidate the mechanism by which PP2A-inhibition alters cisplatin permeability will inform the rational combination of LB100 with other chemotherapeutic agents.

Resistance to chemotherapy represents a significant therapeutic challenge for recurrent MBs that relapse after standard therapy [6]. For patients with recurrent disease, the prognosis is dismal with a 5-year survival of approximately 25% [39]. The ability of LB100 to overcome cisplatin resistance in an *in vitro* model suggests that LB100 could reduce MB chemoresistance clinically. At low concentrations, LB100 enhanced cisplatin-mediated cytotoxicity and abolished acquired cisplatin resistance. Resistant MB cells had diminished accumulation of cisplatin by ICP-MS analysis, via either impaired influx or enhanced efflux, a common mechanism of acquired cisplatin resistance [40]. LB100 more than reversed the deficit in accumulation of cisplatin in resistant cells, leading to cisplatin levels exceeding those in LB100-treated parental wild type cells. Thereby, we provided evidence that the observed PP2A-inhibition mediated enhancement of cisplatin uptake could play an important role in treatment of cisplatin resistant cells by augmenting cisplatin uptake.

In summary, we demonstrated LB100 has potential therapeutic benefit in the treatment of MBs. By itself, it inhibited proliferation and induced significant apoptosis of MB, attenuated MB cell migration and enhanced cisplatin cytotoxicity. We provided evidence that LB100 inhibited STAT3 activation, directly increased cisplatin uptake and overcame cisplatin-resistance *in vitro*. In addition, LB100 exhibited potent *in vivo* anti-neoplastic activity in combination with cisplatin in an intracranial xenograft model. We believe these findings provide strong preclinical support for testing of LB100 as an adjunct to cisplatin in clinical trials for MB.

## MATERIALS AND METHODS

### Reagents and antibodies

The primary antibodies pSTAT3 (TYR705), pSTAT3 (SER727), STAT3 (D3Z2S), c-myc, cyclin D, cleaved caspase-3 (5A1E), cleaved PARP (D64E10), MRP1 (D7O8N), survivin and beta-actin were purchased from Cell Signaling and SNAIL primary antibody from Abcam. Fluorescent-conjugated secondary antibodies for quantitative immunoblotting were purchased from Li-Cor Biosciences. HRP-conjugated secondary antibodies for chemiluminescent immunoblotting were purchased from Cell Signaling. Fluorescent-conjugated secondary antibody for flow cytometry was purchased from Life Technologies. Cisplatin was purchased from Sigma-Aldrich and prepared in aliquot of 10-mmol/L stock solution dissolved in PBS and stored in −20°C [41]. LB100, a water-soluble small-molecule inhibitor of PP2A, was provided by Lixte Biotechnology Holdings, Inc. and was similarly prepared as aliquot of 10-mmol/L stock solution dissolved in PBS and stored in −20°C. Solutions for *in vitro* treatment and *in vivo* injections were diluted from stock solution immediately before administration.

### Cell culture

The human medulloblastoma cell lines, DAOY (HTB 186), D283 (HTB-185) and D341 (HTB-187) were obtained from American Type Culture Collection (ATCC). The medulloblastoma cell lines DAOY and D283 were maintained in complete medium, namely Dulbecco's Modified Eagle Medium (DMEM, PAA) with L-glutamine supplemented with 1 mM sodium pyruvate (PAA), 1% penicillin/streptomycin (Invitrogen) and 10% fetal bovine serum (FBS, Invitrogen). The medulloblastoma cell line D341 Med was maintained in DMEM with L-glutamine supplemented with 1 mM sodium pyruvate, 1% penicillin/streptomycin and 20% FBS.

### Cell viability assay

Cell viability was assessed with XTT Assay (ATCC), which contains tetrazolium salt. 96-well plates were seeded with ∼ 1 × 10^4^ DAOY, 2 × 10^4^ D283 and D341. After overnight culture in complete medium, cells were treated with various concentrations of LB100 and/or cisplatin. The XTT assays were carried out according to the manufacturer's instructions after 48 hours of treatment. Absorbance values were determined at 490 and 650 nanometers on an ELx800 spectrophotometer (BioTek). All the XTT assays were performed in triplicate. To determine whether LB100 could enhance the cytotoxic effect of cisplatin, cells were pretreated with LB100 for 3 hours before the addition of cisplatin. Cells were treated with both drugs for 48 hours. Cell viability was calculated in proportion of untreated control. The 50% inhibitory concentration (IC_50_) values were defined as the drug concentrations required to reduce cell numbers to 50% of control.

### PP2A phosphatase activity assay

MB cells were grown to 80% confluence (for DAOY) and ∼0.5−1 × 10^6^ cells/ml (for D283 and D341) in 100-mm dishes and treated with LB100 as indicated and prepared as described previously [17]. Following treatment for 3 hours, cells were washed twice with cold TBS (pH 7.4) and lysed in RIPA lysis buffer (Thermo Scientific) supplemented with protease inhibitors (Roche) for 30 minutes on ice. Cell lysates were sonicated for 10 seconds then centrifuged at maximum speed for 15 minutes. Supernatants containing 50 ug of total cellular protein were assayed with the PP2A Phosphatase Assay Kit (Millipore) according to the manufacturer's instructions. Experiments were performed in triplicate, and the data are presented as a percentage mean of relative PP2A activity compared with control ± SE.

### Cell migration assay

For the *in vitro* invasion assay DAOY cells were plated in a 2-well cell culture-insert dish (ibidi). About 1.4 × 10^4^ cells were plated on each well of 0.22 cm^2^, separated by a culture insert. After plating, cells were allowed to adhere overnight. The insert was then removed leaving a gap of 500 um between the two wells. The cells were then treated with vehicle control (PBS), 1 uM cisplatin or 1 uM LB100. The culture dish was then placed in a Nikon BioStation and time-lapse wide-field phase contrast images were obtained every 15 minutes for 48 hours. The time it takes for the cells in the two wells to completely invade the 500 um gap was then determined and compared between treatment groups. The images were analyzed using the NIS-Elements Imaging Software (Nikon).

### Apoptosis assay and cell cycle analysis

About 1 × 10^6^ cells were plated in 100-mm dishes (DAOY) or 6-well plates (D283 and D341). After 48-hour treatment, cells were then fixed and permeabilized using the Click-iT EdU Plus flow cytometry assay kit (Thermo Scientific) according to the manufacturer's instructions. For apoptosis-only assay, after fixation and permeabilization, cells were incubated with cleaved Caspase-3 (cC3) and cleaved PARP (cPARP) antibodies for one hour then stained with appropriate secondary antibody for 30 minutes. Finally cells were incubated in DAPI (1 μg/ml). For cell cycle analysis, cells were pulse labeled with 10 μM EdU for 1.5 hours prior to fixation. Before staining with the above apoptotic markers, the Click-iT reaction was performed per the manufacturer's protocol, followed by staining with cC3 and cPARP antibodies, and counterstaining with DAPI to assay total DNA content per cell. Cells were then analyzed by flow cytometry (MoFlo Astrios cell sorter with Summit acquisition software, Beckman Coulter). Data analysis was completed with Kaluza software (Beckman Coulter). All data are presented as a percentage mean ± SE.

### Immunoblotting analysis

Whole-cell and homogenized tumor tissues were lysed in RIPA buffer (Thermo Scientific), supplemented with Complete Protease Inhibitor Cocktail tablets and PhosStop phosphatase inhibitors (Roche). Total cellular proteins (10–40 μg) were separated on 4–12% NuPage SDS–PAGE gel (Invitrogen) and transferred onto polyvinylidene fluoride (PVDF) membranes (Invitrogen). For immunoblotting with densitometry analysis, membranes were blocked for 1 hr at room temperature in Odyssey blocking buffer (LI-COR Bioscience) and probed overnight with primary antibodies followed by anti-mouse or anti-rabbit fluorescent antibodies in blocking buffer for 1 hour at a 1:10,000 dilution. Following a series of final washes, blots were imaged using the Odyssey^®^ Fc imaging system (Li-Cor Biosciences). Signal quantitation and analysis was performed with the Image Studio software (version 5.x) native to the Odyssey Fc imaging system (Li-Cor Biosciences). For immunoblotting without densitometry analysis, membranes were blocked in 5% (w/v) nonfat milk in TBS-Tween-20 and probed overnight with primary antibodies followed by anti-rabbit or anti-mouse IgG–horseradish peroxidase (HRP)–conjugated secondary antibodies (Cell Signaling) in blocking buffer for 1 hour. Membranes were subsequently incubated in the SuperSignal West Pico Chemiluminescent HRP Substrate (Thermo Scientific) and developed on BioMax XAR film (Kodak).

### STAT3 localization

Immunofluorescent cytochemical staining for STAT3 was performed. Cells were grown in chamber slides with varying concentration of LB100 for 4 hours. Cells were fixed with 2% paraformaldehyde, washed with PBS, permeabilized with 1% Triton X-100, washed again with PBS, and blocked with 1% BSA. Rabbit anti-STAT3 (D3Z2G) antibody (Cell signaling) was added at 1:1000 dilution and incubated for 1.5 hours at room temperature. Cells were washed with 1% BSA and mouse anti-rabbit-FITC antibody (Jackson ImmunoResearch) was added at 1:100 and incubated for 1 hour at room temperature. Nuclei were counterstained with DAPI (Sigma-Aldrich). Coverslips were mounted with VectaShield anti-fade solution (Vector Labs) and slides examined on a Nikon Eclipse CI fluorescent microscope and imaged with a QImaging camera.

### Whole cell platinum measurement by inductively coupled plasma-mass spectrometry (ICP-MS)

About 3 × 10^6^ cells were pelleted and lysed in 500 μl of 1% Triton X-100, 0.1% SDS at room temperature for 30 minutes. A small aliquot was saved to quantitate protein using the BCA protein assay to normalize each sample (Thermo-Pierce). Samples were wet ashed by digesting the lysate in 500 μl of concentrated trace metal nitric acid (JT Baker INSTRA-ANALYZED Plus) for 30 minutes in boiling water. Lysates were cooled to room temperature and 500 μl of 30% hydrogen peroxide was added (Fisher Scientific) incubated for 30 minutes in boiling water. Samples were diluted 1:10 with ultrapure 18 mΩ water to decrease the final nitric acid concentration to 3%. Samples were analyzed by ICP-MS using the NexION 350D (Perkin Elmer) equipped with the microFAST system (Elemental Scientific Inc.). Nebulizer gas flow, torch alignment, and Quadrupole Ion Deflector (QID) were optimized daily to pass the standard performance check prior to analysis. Holmium was used as the internal standard and added inline (stock concentration 10 ppb) to each sample and standard. A 1 ppb standard curve (0.1, 0.5, 1.0 ppb platinum, Perkin Elmer) was prepared in 2% nitric acid. Platinum 195 and Holmium 165 were measured in standard mode using peak hopping scan mode with 40 sweeps per reading and 3 replicates for each sample. Dwell times for Platinum 195 were 50 ms and Holmium was 25 ms. Each sample was ran three times to verify concentration. Values were normalized to total protein for each sample.

### Cisplatin resistant DAOY cell line

DAOY cells were given 2–3 day pulses of increasing concentrations of cisplatin. Cells were allowed to recover until about 70% confluence before being pulsed again. This was repeated for a 4 month duration and the highest cisplatin concentration used was 5 uM. A stably resistant cell line (DAOY-CR5P) was generated with a cisplatin IC50 of 7.4 uM compared to 4.5 uM in the parental wild type (DAOY-WT).

### Luciferase-expressing DAOY cell line

Luciferase-expressing cells were generated by transduction of DAOY cells with LV-CMV-FLuc-IRES-GFP lentivirus (MOI 10) to label the cells with Firefly-Luiciferase and GFP (Capital Bioscience). Using FACS, the 10% brightest population in GFP signal was sorted and expanded. Transduced cells were maintained in neomycin selection (500 ug/ml). Prior to implantation, FACS was performed to ensure at least > 80% GFP positive cells.

### Intracranial xenograft model

6–8 week old immunocompromised (IcrTac: ICR-*Prkdc*^scid^) mice from Taconic Bioscience (Hudson, NY) were used in the *in vivo* experiments. Animals were fed animal chow and water ad *libitum*. All animal studies were conducted in accordance with the principles and procedures outlined in the NIH Guide for the Care and Use of Animals and approved by the Animal Care and Use Committee of the National Institute of Health. To implant the tumors, the mouse head was fixated in a stereotactic apparatus. The skin over the skull was cleaned with antimicrobial solution. A longitudinal incision was made between the occiput and forehead. Using a high-speed drill with a burr-tip 0.5 mm in diameter a small burr hole was made to accommodate the needle tip. 3 μl of a thick single cell suspension (2.5 × 10^5^ cells) was injected with a 28 gauge microsyringe (10 μl, Hamilton, NV) perpendicular to the cranial surface into the right cerebellar hemisphere (2 mm to the right of the midline, 2 mm posterior to the lambdoid suture, and 2 mm deep). After implantation the animals were independently monitored daily by the animal facility staff. The animals were euthanized when they displayed neurologic symptoms or lost > 20% of peak body weight.

*In vivo* bioluminescent imaging. Mice were anesthetized in a chamber with an oxygen-enhanced air gas mixture containing 3–5% isofluorane. Animals were then given an ip injection with 150 mg/kg of D-luciferin. After 15 minutes, the animals were imaged using a IVIS 200 imaging station (Caliper Life Sciences). Regions of interest were defined using living image software, and the total photons/s/sr/cm^2^ (photons per second per steradian per square cm) were recorded every 1–2 weeks to monitor tumor growth and therapy response. The tumor growth was measured and the tumor burden was approximated using the total bioluminescence.

### *In vivo* magnetic resonance imaging

All studies were performed on a 9.4 Tesla magnet equipped with a Bruker Avance III console and a 15 cm gradient from Resonance Research. Imaging was performed with a 89 μm quadrature volume coil for excitation (Bruker-Biospin) and a 4 channel mouse head receive array (Bruker-Biospin). Mice were anesthetized in a chamber with an oxygen-enhanced air gas mixture containing 3–5% isofluorane and then placed supine on a plastic cradle. A custom-built transmit-receive birdcage mouse-head coil was used to acquire T1-weighted images. Multi-slice data was acquired using the manufacturer's FLASH sequence. Imaging parameters were TR/TE = 208/6ms, Field of View = 20 mm, number of averages = 4, imaging matrix = 128 × 128. Pre- and post-contrast images were obtained. IV contrast was injected through a tail vein catheterized with a 27–30 gauge needle.

### Histological characterization

At the endpoint of the survival study, animals were euthanized using CO_2_ inhalation. The cerebellum was excised from the cranium and then formalin-fixed and sectioned (10 μm). Hematoxylin and eosin (H & E) staining was performed. Slides were reviewed by a board certified neuropathologist in a blinded fashion.

### Statistical analysis

The two-sided Student's *t*-test was applied to determine statistical significance between two groups. Ordinary one-way ANOVA test was used for comparison between more than two groups. *p* ≤ 0.05 (*), was considered as statistically significant. Values stated within text and figures represent mean ± standard. Statistics were performed on results from at least two independent replicates.

## References

[1] Dolecek TA, Propp JM, Stroup NE, Kruchko C (2012). CBTRUS statistical report: primary brain and central nervous system tumors diagnosed in the United States in 2005–2009. Neuro Oncol.

[2] Packer RJ, Zhou T, Holmes E, Vezina G, Gajjar A (2013). Survival, secondary tumors in children with medulloblastoma receiving radiotherapy and adjuvant chemotherapy: results of Children's Oncology Group trial A9961. Neuro-oncology.

[3] Gottardo NG, Hansford JR, McGlade JP, Alvaro F, Ashley DM, Bailey S, Baker DL, Bourdeaut F, Cho YJ, Clay M, Clifford SC, Cohn RJ, Cole CH (2014). Medulloblastoma Down Under 2013: a report from the third annual meeting of the International Medulloblastoma Working Group. Acta Neuropathol.

[4] Fossati P, Ricardi U, Orecchia R (2009). Pediatric medulloblastoma: toxicity of current treatment and potential role of protontherapy. Cancer Treat Rev.

[5] Frange P, Alapetite C, Gaboriaud G, Bours D, Zucker JM, Zerah M, Brisse H, Chevignard M, Mosseri V, Bouffet E, Doz F (2009). From childhood to adulthood: long-term outcome of medulloblastoma patients. The Institut Curie experience (1980–2000). J Neurooncol.

[6] Dunkel IJ, Gardner SL, Garvin JH, Goldman S, Shi W, Finlay JL (2010). High-dose carboplatin, thiotepa, and etoposide with autologous stem cell rescue for patients with previously irradiated recurrent medulloblastoma. Neuro Oncol.

[7] Kool M, Korshunov A, Remke M, Jones DT, Schlanstein M, Northcott PA, Cho YJ, Koster J, Schouten-van Meeteren A, van Vuurden D, Clifford SC, Pietsch T, von Bueren AO (2012). Molecular subgroups of medulloblastoma: an international meta-analysis of transcriptome, genetic aberrations, and clinical data of WNT, SHH, Group 3, and Group 4 medulloblastomas. Acta Neuropathol.

[8] Samkari A, White JC, Packer RJ (2015). Medulloblastoma: toward biologically based management. Semin Pediatr Neurol.

[9] Hong CS, Ho W, Zhang C, Yang C, Elder JB, Zhuang Z (2015). LB100, a small molecule inhibitor of PP2A with potent chemo- and radio-sensitizing potential. Cancer Biol Ther.

[10] Perrotti D, Neviani P (2013). Protein phosphatase 2A: a target for anticancer therapy. Lancet Oncol.

[11] Mumby M (2007). PP2A: unveiling a reluctant tumor suppressor. Cell.

[12] Lu J, Kovach JS, Johnson F, Chiang J, Hodes R, Lonser R, Zhuang Z (2009). Inhibition of serine/threonine phosphatase PP2A enhances cancer chemotherapy by blocking DNA damage induced defense mechanisms. Proc Natl Acad Sci U S A.

[13] Vincent Chung AM, Fadi Braiteh, Carlos Becerra, Donald Richards (2015). Phase I Study of LB-100 With Docetaxel in Solid Tumors.

[14] Chang KE, Wei BR, Madigan JP, Hall MD, Simpson RM, Zhuang Z, Gottesman MM (2015). The protein phosphatase 2A inhibitor LB100 sensitizes ovarian carcinoma cells to cisplatin-mediated cytotoxicity. Mol Cancer Ther.

[15] Zhang C, Hong CS, Hu X, Yang C, Wang H, Zhu D, Moon S, Dmitriev P, Lu J, Chiang J, Zhuang Z, Zhou Y (2015). Inhibition of protein phosphatase 2A with the small molecule LB100 overcomes cell cycle arrest in osteosarcoma after cisplatin treatment. Cell Cycle.

[16] Bai XL, Zhang Q, Ye LY, Hu QD, Fu QH, Zhi X, Su W, Su RG, Ma T, Chen W, Xie SZ, Chen CL, Liang TB (2014). Inhibition of protein phosphatase 2A enhances cytotoxicity and accessibility of chemotherapeutic drugs to hepatocellular carcinomas. Mol Cancer Ther.

[17] Gordon IK, Lu J, Graves CA, Huntoon K, Frerich JM, Hanson RH, Wang X, Hong CS, Ho W, Feldman MJ, Ikejiri B, Bisht K, Chen XS (2015). Protein Phosphatase 2A Inhibition with LB100 Enhances Radiation-Induced Mitotic Catastrophe and Tumor Growth Delay in Glioblastoma. Mol Cancer Ther.

[18] Lv P, Wang Y, Ma J, Wang Z, Li JL, Hong CS, Zhuang Z, Zeng YX (2014). Inhibition of protein phosphatase 2A with a small molecule LB100 radiosensitizes nasopharyngeal carcinoma xenografts by inducing mitotic catastrophe and blocking DNA damage repair. Oncotarget.

[19] Wei D, Parsels LA, Karnak D, Davis MA, Parsels JD, Marsh AC, Zhao L, Maybaum J, Lawrence TS, Sun Y, Morgan MA (2013). Inhibition of protein phosphatase 2A radiosensitizes pancreatic cancers by modulating CDC25C/CDK1 and homologous recombination repair. Clin Cancer Res.

[20] Yu H, Lee H, Herrmann A, Buettner R, Jove R (2014). Revisiting STAT3 signalling in cancer: new and unexpected biological functions. Nat Rev Cancer.

[21] Cattaneo E, Magrassi L, De-Fraja C, Conti L, Di Gennaro I, Butti G, Govoni S (1998). Variations in the levels of the JAK/STAT and ShcA proteins in human brain tumors. Anticancer Res.

[22] Schaefer LK, Ren Z, Fuller GN, Schaefer TS (2002). Constitutive activation of Stat3alpha in brain tumors: localization to tumor endothelial cells and activation by the endothelial tyrosine kinase receptor (VEGFR-2). Oncogene.

[23] Yang F, Van Meter TE, Buettner R, Hedvat M, Liang W, Kowolik CM, Mepani N, Mirosevich J, Nam S, Chen MY, Tye G, Kirschbaum M, Jove R (2008). Sorafenib inhibits signal transducer and activator of transcription 3 signaling associated with growth arrest and apoptosis of medulloblastomas. Mol Cancer Ther.

[24] Craveiro RB, Ehrhardt M, Holst MI, Pietsch T, Dilloo D (2014). In comparative analysis of multi-kinase inhibitors for targeted medulloblastoma therapy pazopanib exhibits promising *in vitro* and *in vivo* efficacy. Oncotarget.

[25] Xiao H, Bid HK, Jou D, Wu X, Yu W, Li C, Houghton PJ, Lin J (2015). A novel small molecular STAT3 inhibitor, LY5, inhibits cell viability, cell migration, and angiogenesis in medulloblastoma cells. J Biol Chem.

[26] Yang F, Jove V, Xin H, Hedvat M, Van Meter TE, Yu H (2010). Sunitinib induces apoptosis and growth arrest of medulloblastoma tumor cells by inhibiting STAT3 and AKT signaling pathways. Mol Cancer Res.

[27] Chang CJ, Chiang CH, Song WS, Tsai SK, Woung LC, Chang CH, Jeng SY, Tsai CY, Hsu CC, Lee HF, Huang CS, Yung MC, Liu JH (2012). Inhibition of phosphorylated STAT3 by cucurbitacin I enhances chemoradiosensitivity in medulloblastoma-derived cancer stem cells. Childs Nerv Syst.

[28] Yang MY, Lee HT, Chen CM, Shen CC, Ma HI (2014). Celecoxib suppresses the phosphorylation of STAT3 protein and can enhance the radiosensitivity of medulloblastoma-derived cancer stem-like cells. Int J Mol Sci.

[29] Woetmann A, Nielsen M, Christensen ST, Brockdorff J, Kaltoft K, Engel AM, Skov S, Brender C, Geisler C, Svejgaard A, Rygaard J, Leick V, Odum N (1999). Inhibition of protein phosphatase 2A induces serine/threonine phosphorylation, subcellular redistribution, and functional inhibition of STAT3. Proc Natl Acad Sci U S A.

[30] Mitsuhashi S, Shima H, Tanuma N, Sasa S, Onoe K, Ubukata M, Kikuchi K (2005). Protein phosphatase type 2A, PP2A, is involved in degradation of gp130. Mol Cell Biochem.

[31] Mandal T, Bhowmik A, Chatterjee A, Chatterjee U, Chatterjee S, Ghosh MK (2014). Reduced phosphorylation of Stat3 at Ser-727 mediated by casein kinase 2 - protein phosphatase 2A enhances Stat3 Tyr-705 induced tumorigenic potential of glioma cells. Cell Signal.

[32] Snuderl M, Batista A, Kirkpatrick ND, Ruiz de Almodovar C, Riedemann L, Walsh EC, Anolik R, Huang Y, Martin JD, Kamoun W, Knevels E, Schmidt T (2013). Targeting placental growth factor/neuropilin 1 pathway inhibits growth and spread of medulloblastoma. Cell.

[33] Triscott J, Lee C, Foster C, Manoranjan B, Pambid MR, Berns R, Fotovati A, Venugopal C, O'Halloran K, Narendran A, Hawkins C, Ramaswamy V, Bouffet E (2013). Personalizing the treatment of pediatric medulloblastoma: Polo-like kinase 1 as a molecular target in high-risk children. Cancer Res.

[34] Liu WH, Chen MT, Wang ML, Lee YY, Chiou GY, Chien CS, Huang PI, Chen YW, Huang MC, Chiou SH, Shih YH, Ma HI (2015). Cisplatin-selected resistance is associated with increased motility and stem-like properties via activation of STAT3/Snail axis in atypical teratoid/rhabdoid tumor cells. Oncotarget.

[35] Cole SP (2014). Multidrug resistance protein 1 (MRP1, ABCC1), a "multitasking" ATP-binding cassette (ABC) transporter. J Biol Chem.

[36] Pizem J, Cort A, Zadravec-Zaletel L, Popovic M (2005). Survivin is a negative prognostic marker in medulloblastoma. Neuropathol Appl Neurobiol.

[37] Northcott PA, Shih DJ, Peacock J, Garzia L, Morrissy AS, Zichner T, Stutz AM, Korshunov A, Reimand J, Schumacher SE, Beroukhim R, Ellison DW, Marshall CR (2012). Subgroup-specific structural variation across 1,000 medulloblastoma genomes. Nature.

[38] Kaufhold S, Bonavida B (2014). Central role of Snail1 in the regulation of EMT and resistance in cancer: a target for therapeutic intervention. J Exp Clin Cancer Res.

[39] Millard NE, De Braganca KC (2015). Medulloblastoma. J Child Neurol.

[40] Shen DW, Pouliot LM, Hall MD, Gottesman MM (2012). Cisplatin resistance: a cellular self-defense mechanism resulting from multiple epigenetic and genetic changes. Pharmacol Rev.

[41] Hall MD, Telma KA, Chang KE, Lee TD, Madigan JP, Lloyd JR, Goldlust IS, Hoeschele JD, Gottesman MM (2014). Say no to DMSO: dimethylsulfoxide inactivates cisplatin, carboplatin, and other platinum complexes. Cancer Res.

